# A population-wide gene-environment interaction study on how genes, schools, and residential areas shape achievement

**DOI:** 10.1038/s41539-022-00145-8

**Published:** 2022-10-27

**Authors:** Rosa Cheesman, Nicolai T. Borgen, Torkild H. Lyngstad, Espen M. Eilertsen, Ziada Ayorech, Fartein A. Torvik, Ole A. Andreassen, Henrik D. Zachrisson, Eivind Ystrom

**Affiliations:** 1 PROMENTA Research Center, Department of Psychology, University of Oslo, Oslo, Norway; 2 Department of Special Needs Education, Faculty of Educational Sciences, University of Oslo, Oslo, Norway; 3 Department of Sociology & Human Geography, University of Oslo, Oslo, Norway; 4 Centre for Fertility and Health, Norwegian Institute of Public Health, Oslo, Norway; 5 NORMENT Centre, Division of Mental Health and Addiction, Oslo University Hospital & Institute of Clinical Medicine, University of Oslo, Oslo, Norway; 6 Department of Mental Disorders, Norwegian Institute of Public Health, Oslo, Norway

**Keywords:** Human behaviour, Human behaviour, Sociology, Education

## Abstract

A child’s environment is thought to be composed of different levels that interact with their individual genetic propensities. However, studies have not tested this theory comprehensively across multiple environmental levels. Here, we quantify the contributions of child, parent, school, neighbourhood, district, and municipality factors to achievement, and investigate interactions between polygenic indices for educational attainment (EA-PGI) and environmental levels. We link population-wide administrative data on children’s standardised test results, schools and residential identifiers to the Norwegian Mother, Father, and Child Cohort Study (MoBa), which includes >23,000 genotyped parent-child trios. We test for gene-environment interactions using multilevel models with interactions between EA-PGI and random effects for school and residential environments (thus remaining agnostic to specific features of environments). We use parent EA-PGI to control for gene-environment correlation. We found an interaction between students’ EA-PGI and schools suggesting compensation: higher-performing schools can raise overall achievement without leaving children with lower EA-PGI behind. Differences between schools matter more for students with lower EA-PGI, explaining 4 versus 2% of the variance in achievement for students 2 SD below versus 2 SD above the mean EA-PGI. Neighbourhood, district, and municipality variation contribute little to achievement (<2% of the variance collectively), and do not interact with children’s individual EA-PGI. Policy to reduce social inequality in achievement in Norway should focus on tackling unequal support across schools for children with difficulties.

## Introduction

Individual differences in school achievement are shaped by a complex interplay between genes and environments. Theories of child development such as the bioecological model emphasise that the environment is composed of multiple levels, including not only on the family, but also schools, neighbourhoods, and wider society, institutions, and culture^1^. Although a child’s immediate surroundings where social interactions and formal learning take place (family and school) are theorised to be most important, more distal factors (in neighbourhoods, and societal institutions) should also matter for achievement^2–4^. Empirical evidence on the relative importance of these levels remains scarce because comprehensive measurements of any one of them are difficult, and, importantly, because it is challenging to separate effects of these intercorrelated levels. For example, neighbourhood effects may be diminished after schools are controlled for^5^.

Gene-environment interaction research focuses on the environmental contingency of genetic effects (and vice versa)^6^. An influential interaction hypothesis is that disadvantage (e.g., environments with low intellectual and financial resources) suppresses genetic influence on cognitive development, whereas advantage allows genetic differences to be expressed. This model, often referred to as the Scarr-Rowe interaction^7^, has led to the notion that high heritability is a marker of an advantageous environment^8^. However, the model has not found consistent support in empirical data on achievement and cognition. Many, but not all^9^ U.S. twin studies have found that genetic influences are stronger in higher-socioeconomic status families, whereas European and Australian studies have found null or opposite results^10^. A recent twin study found that heritability estimates for achievement were invariant across levels of parental socioeconomic status in Norwegian, German, and U.S. samples, but in a Swedish dataset, heritability estimates were stronger in more disadvantaged families^11^. Genomic studies have also found largely null results. Polygenic indices (PGI) – which measure individual-level genetic propensity for traits – generally appear not to interact with the environment in analyses of achievement outcomes. This holds even when modelling numerous PGI and family environmental measures, including chaos at home, parental job loss, parental educational attainment and income^12–14^. It is debated whether the Scarr-Rowe interaction applies to the full range of environmental experience or only in extremely deprived circumstances^15^.

Three key factors limit the utility of this prior evidence on gene-environment interaction and the Scarr-Rowe model. First, the scope of the environments considered has generally been narrow, focusing on familial ‘micro-environments’^16^ such as parental education. Several studies suggest that gene-environment interactions beyond the family warrant further research. Genetic influence on achievement (at least in the U.S.) appears to be stronger in the presence of higher neighbourhood income, higher school quality, and higher quality teachers^17–19^, in line with the Scarr-Rowe model. However, we are not aware of any studies that have *simultaneously* considered multiple relevant environmental levels, as the bioecological model would recommend. If effects of intercorrelated contexts are not distinguished, educational interventions could be misdirected.

Second, studies have overwhelmingly tested interactions of individual genetic differences with whatever *specific* environmental measures are available. This strategy, whilst allowing any relevant interactive contexts to be pinpointed, fails to capture the total importance of interactions, including with unmeasured or latent environments. Two studies used multilevel modelling to estimate the total magnitude of interactions between schools and PGI^20,21^. The multilevel modelling approach is useful because it is agnostic to specific features of environmental levels (which are challenging to identify and measure accurately) but indicates whether investigation of specific measured environments at different levels is justified.

The third difficulty is accounting for the endogeneity of social contexts that stems from gene-environment correlation^22^. Indeed, parents are known to select schools and residential areas for their offspring. When selection is based on heritable characteristics, *passive* gene-environment correlation can occur, whereby offspring inherit correlated environments and genetic propensities from their parents^6,23^. When children themselves select into environments (e.g., test scores gain them places at selective schools), *active* gene-environment correlation can occur. Estimating gene-environment interactions in the presence of gene-environment correlation can lead to false positive results^24,25^. Here, we control for gene-environment correlation using the random nature of parent-to-child genetic inheritance. Controlling for parental genotypes, effects of offspring genotype are solely due to random segregation of genetic material during meiosis and cannot stem from passive gene-environment correlation and other confounding effects.

In sum, previous studies have not estimated the full magnitude of gene-environment interactions due to narrow focus on family-level environments. Where interactions have been found, they may be confounded by environmental factors on different levels to those measured, and/or by gene-environment correlation. This suggests the need for a more comprehensive research strategy with a wider approach to children’s social contexts.

Here, we use a sample of >23,000 parent-child trios residing across Norway to quantify how school achievement is influenced by interactions between students’ educational attainment PGI (EA-PGI) and *multiple* levels of social context. We use multilevel models to estimate *total* interactions of PGI with schools, neighbourhoods, districts, and municipalities, while remaining agnostic to specific features of these contexts. We use *within-family* EA-PGI (child PGI adjusted for parental PGI) to control for passive gene-environment correlation. Having characterised total interaction effects, we investigate whether interactions are explained by measured sociodemographic features or remain to be identified. The bioecological model leads to the hypothesis that interactions exist across environmental levels, but are smaller at more distal levels (e.g., municipalities). The Scarr-Rowe model predicts that PGI effects are weaker in less advantaged environments (e.g., schools with lower average achievement). Norway is a relatively egalitarian country, where almost all children attend their local public school, and social differences are minimised by redistributive policies^26,27^. However, small average effects of schools and residential areas on education may conceal a greater impact for students with certain individual characteristics. Our gene-environment interaction strategy aims to characterise these children, and ultimately to identify which environments work best for them.

## Results

We integrated genetic data from the Norwegian Mother, Father, and Child Cohort Study (MoBa) with administrative data on young people’s standardised national test results in maths, reading and English in grades 5, 8 and 9, and their school, neighbourhood, district, and municipality membership. Our models included 23,471 students with non-missing data for achievement, their educational attainment polygenic indices (EA-PGI), school and residential identifiers, and parental variables (EA-PGI, educational attainment and income). Participating students attended 2578 schools and resided in 408 municipalities, 1440 districts, and 7700 neighbourhoods. There were on average 11 students per school (range 1-66, median 8), 57.3 per municipality (range 1-1643; median 20), 16 per district (range 1-268, median 11), and three per neighbourhood (range 1-51, median 2). Residents of each neighbourhood live in the same district and municipality, but those living in the same neighbourhood do not always attend the same school and vice versa (in 84% of the neighbourhoods, all students attended the same school; students attending the same school were from five different neighbourhoods on average). See Supplementary Table 1 for descriptive statistics of analysis variables.

**Fig. 1:**
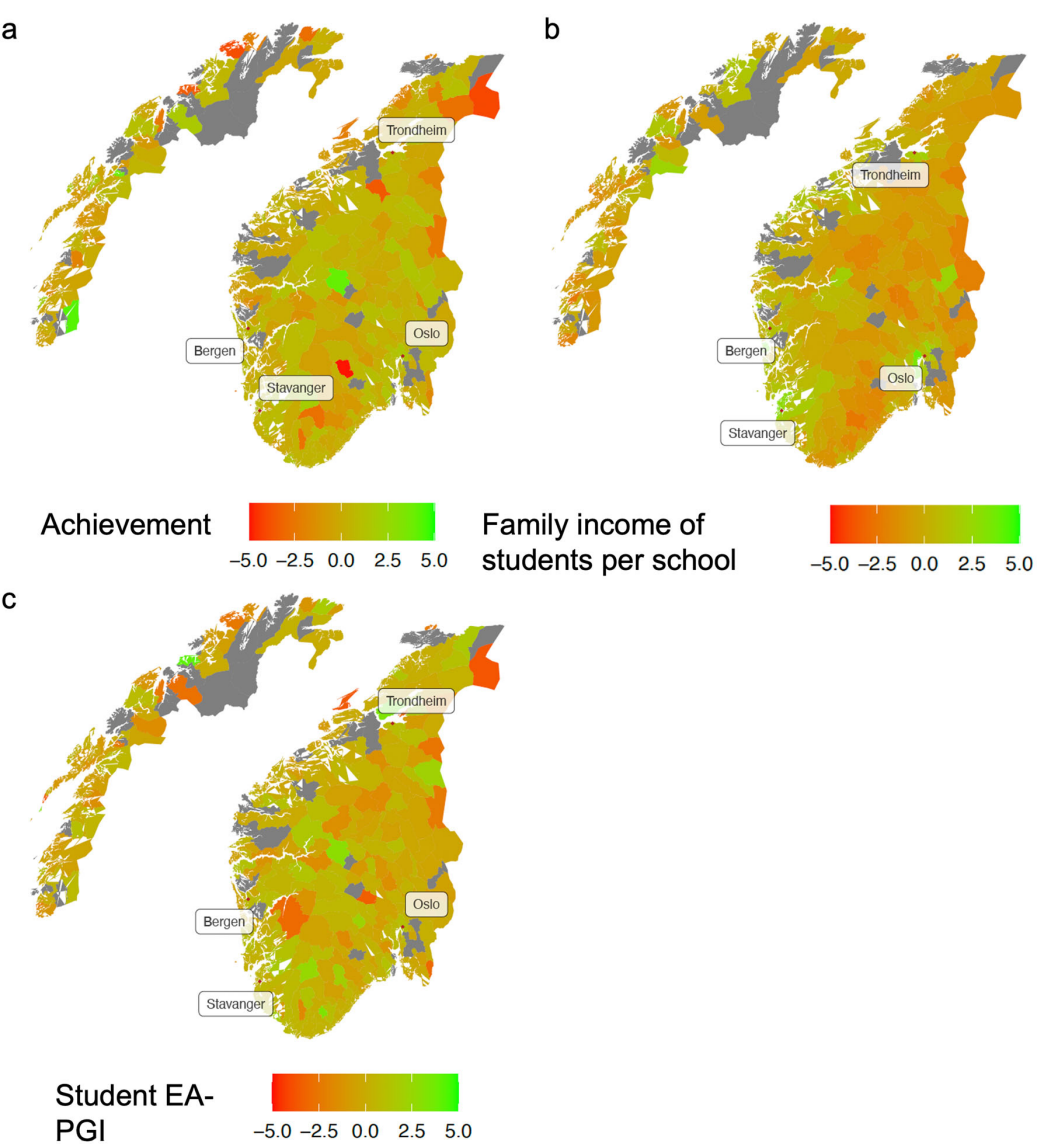
Norwegian municipalities, coloured by average values of variables for students in our analytic sample (a = achievement; b = family income; c = student EA-PGI). Notes: We aggregated to the broader municipality level due to the anonymity of the school and neighbourhood identifiers. Maps are based on grade 5 variables and residential identifiers. Grey= no participants resided in that municipality. Some municipalities are more sparsely populated, such that the depth of colour only reflects one or two participants. This does not hold for school-level family income (1B), which was based on the average income of *all* parents of peers attending participants’ schools, not only other study participants’ parents.

We first display the municipality-level averages of three study variables (Fig. 1). The maps highlight the population-wide coverage of our genetic and socioeconomic data: only a few municipalities were not covered by study participants. Figure 1a and c indicate some municipality-level variation in student achievement and polygenic indices for educational attainment (EA-PGI), respectively. Figure 1b shows clearer patterning, reflecting that the parents of children attending schools in and around major cities (Oslo, Bergen, Trondheim, Stavanger, Tromsø) have the highest incomes (strongest green colour).

### Interactions between students’ EA-PGI and their schools (but not residential areas)

To test for gene-environment interactions, we compared the fit of multilevel models with and without varying EA-PGI effects on achievement (a composite of maths, reading and English subjects) between schools and between residential areas. We started with a simple fixed-effects model regressing student achievement on the EA-PGI and covariates. We then tested if model fit improved upon inclusion of random intercepts (i.e., main effects of schools and residential areas), and then of random slopes (i.e., interactions of student EA-PGI with schools and residential areas) (see Table 1). This multilevel modelling approach provides estimates of total latent effects of school and residential levels, without having to measure specific environments. Since parental EA-PGI were adjusted for in all models, student EA-PGI effects reflect less-biased *within-family* genetic influences, disentangled from environments selected by parents. Note however that some bias may remain since PGI were calculated using SNP weights from between-family GWAS, due to the lack of well-powered within-family GWAS excluding MoBa.

**Table 1:** Model-fitting procedure.

Model	Fixed effects	Random effects
1. Base: EA-PGI effects	Child EA-PGI Parent EA-PGI, income, education Grade	Individual child
2. School/area effects	Child EA-PGI Parent EA-PGI, income, education Grade	Individual child a) School b) + Neighbourhood c) + District d) + Municipality
3. EA-PGI-by-school/area interactions	Child EA-PGI Parent EA-PGI, income, education Grade	Individual child a) Child EA-PGI | School b) + Child EA-PGI | Neighbourhood c) + Child EA-PGI | District d) + Child EA-PGI | Municipality
4. Accounting for school effects	Child EA-PGI Parent EA-PGI, income, education 5 school measures Grade	Individual child Child EA-PGI | School
5. Accounting for EA-PGI by-school interactions	Child EA-PGI Parent EA-PGI, income, education 5 school measures Each school measure * Child EA-PGI Grade	Individual child Child EA-PGI | School

Note that | indicates a random slope effect. Note that 10 principal components reflecting parental genetic ancestry were included as fixed effects in all models (not shown in Table 1).

The best-fitting model – Model 3a in Table 1 – included random slopes and intercepts for schools, but only random intercepts (not slopes) for residential areas (see Supplementary Table 2 for fit statistics and Supplementary Table 3 for results). This indicates that the effects of students’ EA-PGI depend on schools, but not on neighbourhoods, districts, or municipalities, which only have small main effects on achievement. The variance explained in achievement by residential areas was 1% for municipalities, 1% for neighbourhoods, and <1% for districts (see Supplementary Table 4a for intraclass correlations).

**Fig. 2:**
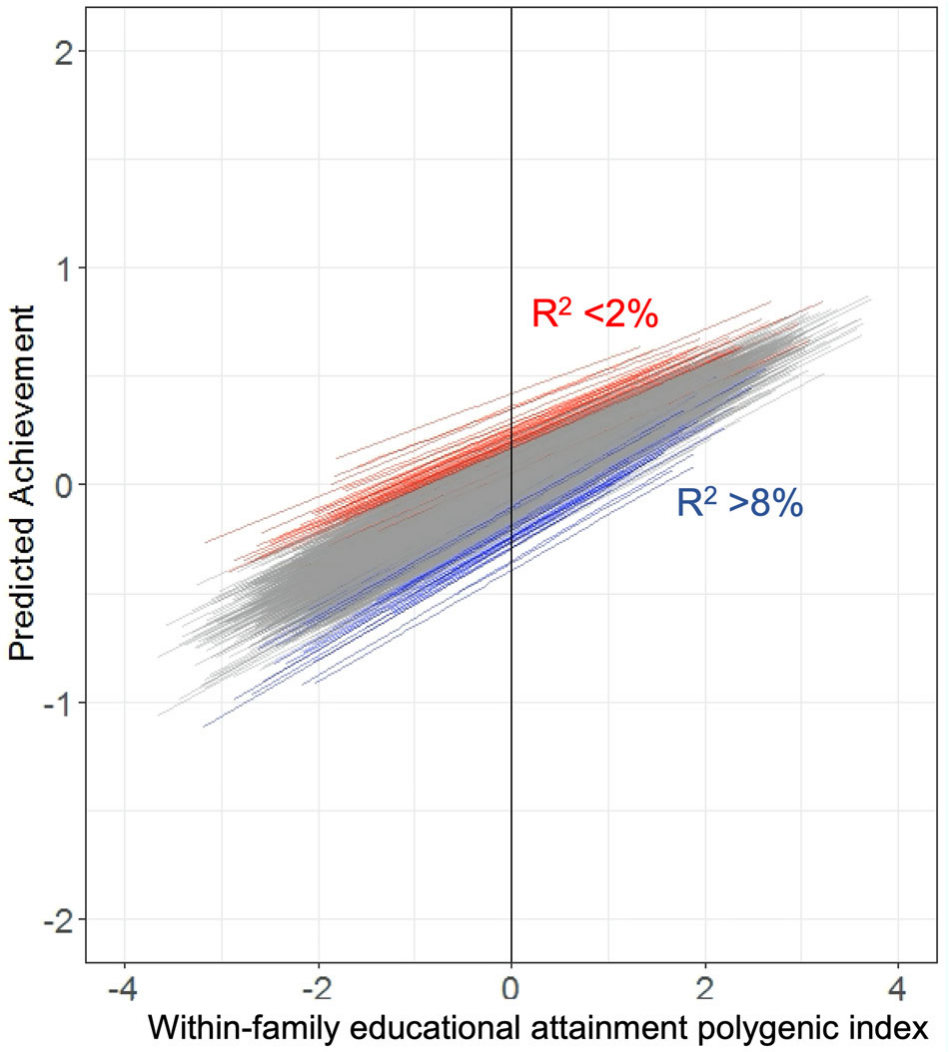
School-specific associations between the within-family EA-PGI and achievement. In red are regression lines for the 2.5% schools in which within-family EA-PGI effects are weakest; in blue are the 2.5% of schools where PGI effects are strongest; R^2^ = variance explained in achievement. The within-family EA-PGI has a weaker effect in schools where average student achievement is higher. School differences in achievement are wider among students with lower EA-PGI. Note that the sample size was 23471 children attending 2578 schools.

Three main aspects of the EA-PGI-by-school interaction are visualised in Fig. 2, which shows school-specific EA-PGI effects on achievement. First, the mean effect of the EA-PGI on achievement is 0.22 but there is variation around this average slope between schools (standard deviation of slopes = 0.034). In the 2.5% of schools with the weakest effects (red lines), the effect of students’ EA-PGI is <15% of an SD (i.e., 0.22–1.96*0.034), whereas in the 2.5% of schools with the strongest effects (blue lines), EA-PGI have effects of >29% of an SD. The variance explained by the EA-PGI is therefore more than four times higher than in the former group of schools (~8% versus 2%).

Second, the slope-intercept correlation was negative, meaning that variation in students’ EA-PGI is less influential for achievement in the schools with overall higher student achievement. This is shown in Fig. 2 by the higher positions (intercepts) of the red lines (weakest slopes) compared to blue lines (strongest slopes).

**Fig. 3:**
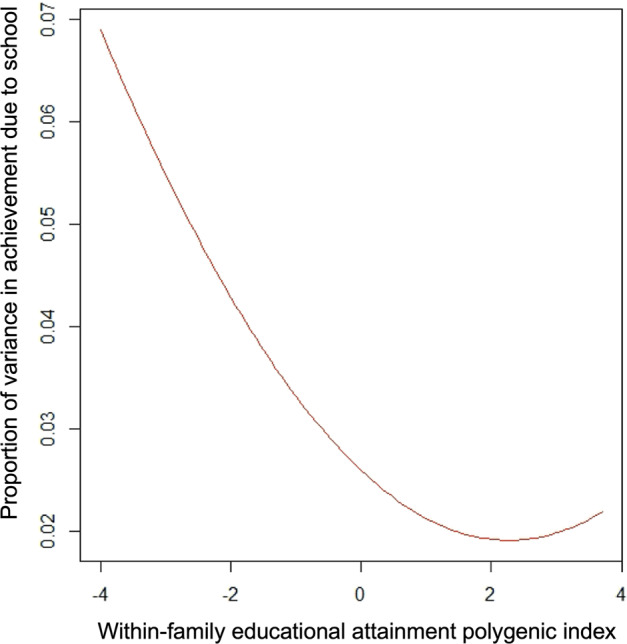
School effects on achievement vary across values of the within-family EA-PGI. For students with within-family EA-PGI that are 2 SD below the mean, schools explain ~4% of the phenotypic variance, whereas for students with PGI 2 SD above the mean, schools explain ~2%.

Third, the interaction also means that the effect of school on student achievement varies according to student EA-PGI. The regression lines in Fig. 2 are fanned out at lower values of the EA-PGI, and taper in as EA-PGI increases. This demonstrates how schools make more of a difference to the achievements of students with lower EA-PGI. In contrast, for students with higher EA-PGI, achievements are more similar regardless of the school. Figure 3 shows how the effect of school on achievement declines with increasing student EA-PGI, with schools explaining 4% of the achievement variance among students with EA-PGI 2 SD below the mean, but 2% for those with EA-PGI 2 SD above the mean (see Supplementary Table 3 for calculations).

Importantly, this best-fitting model included strict controls for selection into schools. Controlling for parental EA-PGI reduces the link between student EA-PGI and schools by removing pathways from parental genotype to student achievement (which includes social selection). Indeed, the within-family child PGI shows no school-level clustering (Supplementary Table 4b). Moreover, the interaction captures genetic interplay with school-level effects, where school effects are net of family social background (parental income and education, and parental EA-PGI), and all latent neighbourhood, district, and municipality level variation in achievement.

To explore whether the gene-environment interaction was driven by a particular school subject, we estimated the best-fitting model (3a) separately for maths, reading, and English rather than for the achievement composite. Results indicate that the interaction involves maths and reading more than English. The standard deviation of slopes for the EA-PGI effect between schools was 0.035 for maths, 0.027 for reading, and 0.004 for English.

### No interactions between school sociodemographic measures and student EA-PGI

To complement the multilevel analyses that are agnostic to the school factors that interact with students’ within-family EA-PGI, we explored whether five school-level covariates (tested simultaneously) explained the interaction. These were school average parental education, average parental income, proportion of nonwestern immigrants, and Gini indices of inequality in parental income and education at each school. None of these measures of school sociodemographics can explain why genetic effects differ in strength between schools: the variance in slopes was not attenuated by including the covariates, and covariate-PGI interactions did not improve model fit. See Supplementary Table 3b for results and Supplementary Table 2 for fit statistics.

## Discussion

We investigated gene-environment interactions for educational achievement, integrating genetic, school, and residential information on >23,000 families living across Norway. By including multiple levels of environmental context, our study is more comprehensive than previous efforts. We found evidence for an interaction between students’ educational attainment polygenic indices (EA-PGI) and their schools, even in our strict within-family genetic design, which essentially randomises students to schools. Higher-performing schools compensate for lower EA-PGI, such that genetic effects are weaker in these schools. Surprisingly for an egalitarian context, social differences between schools matter more for achievement among students with lower EA-PGI. Measures of school sociodemographics cannot explain the observed PGI-school interaction. Residential environments (neighbourhoods, districts, and municipalities) contribute little to variation in achievement and do not interact with students’ EA-PGI. This social-genetic approach contributes to the goal of identifying which learning environments work for whom.

The interaction identified here suggests that effects of students’ EA-PGI and of schools on achievement in Norway cannot be interpreted independently. The within-family EA-PGI effect varies between schools, with PGI differences among children being less salient in schools where overall performance is higher. This complements evidence from the U.S. that higher-status schools buffer students with lower EA-PGI from dropping out of advanced mathematics classes^28^. We also replicated their results in that there was a particularly strong PGI by school interaction for maths compared to reading and English. In future it will be important to identify specific school factors that can minimise the consequences of genetic risks. These results go against the Scarr-Rowe model, because effects of starting differences in genetic endowment are not suppressed but magnified in the less advantaged environments. However, in a different context without Norway’s resource redistribution to ensure high‐quality and universally available education (such as that of the original Scarr-Rowe study), genetic effects might not be strongest in the best-performing schools.

Importantly, the interaction also reveals that the lower the student EA-PGI, the greater the variation in achievement created by schools. Given that almost all Norwegian students attend public school, it is surprising to observe this school-driven social inequality, concentrated among those who may need support the most. This holds even after strict controls for passive gene-environment correlation, family socioeconomic background and residential area. Policymakers may want to focus on finding ways to equalise opportunities between schools for students who are equally low on the EA-PGI distribution. Our finding also suggests that social scientists should consider individual differences when estimating school effects. Omnibus estimates, which are small in many studies^27,29^, conceal a greater importance of school for students with lower EA-PGI.

The observed interaction between students’ EA-PGI and their schools is latent since our multilevel models are agnostic to school characteristics. This provides necessary justification for investigating specific school factors driving interactions. However, none of our five measures of school sociodemographics appear to be involved. If we had relied on a measured-environment approach, the gene-environment interaction could not have been detected. The gap between our finding of latent interactions, and the negligible contribution of measured covariates, highlights the need to better characterise aspects of Norwegian schools that change the role of children’s individual genetic differences. Although factors such as class size may have small main effects on achievement^30^, they could still exert important influence in interaction with genetic differences between children. Our approach offers a framework within which to test the interactive roles of school characteristics.

As proposed by the bioecological model, we find that more distal environmental levels (neighbourhoods, districts, and municipalities) are less important for student achievement than schools are. However, unlike the bioecological model, we observed that the total latent variance explained by residential areas is small (though statistically significant), and area effects do not interact with children’s individual genetic differences. This could be because academic skills in reading and mathematics are the direct targets of instruction in schools but not residential areas, making schools the level at which individual proclivities for educational success are amplified or minimised. Practically, our finding that Norwegian residential areas do not vary considerably in ways that affect children’s school performance (e.g. all neighbourhood differences in our sample explain just 0.5% of the variance) suggests that identifying residential factors as intervention targets might not greatly reduce achievement differences of social origin. Nonetheless, the results do not diminish the importance of residential areas. For example, municipalities are essential for providing educational services and allocating resources, and regional inequalities may adversely affect many other life outcomes such as later educational attainment and physical health.

This study is subject to limitations. First, the generalisability of our results is limited because only participants of European ancestries were included. Moreover, despite the near complete coverage of achievement, school and residential identifiers from administrative records, results could be affected by non-random participation in the MoBa cohort study. Second, current EA-PGI do not capture the full genetic component of education, so our analyses do not inform about the total magnitude of genetic interactions with schools. Third, EA-PGI are based on information pooled across many contexts, so by design might not reflect the portion of the heritability of educational attainment that is most sensitive to differences between schools and residential areas. Future research could adopt a less strict test for gene-environment interaction by using PGI for environmental sensitivity^31^ within our multilevel framework. Finally, while we control for passive gene-environment using parental EA-PGI, children’s own genetic propensities could theoretically still influence their school attendance. However, within-family EA-PGI are not clustered in schools. The absence of selective elementary and middle schools in Norway is also reassuring, in contrast to the United Kingdom, where exam differences between selective and non-selective schools primarily reflect heritable characteristics involved in admission^32^.

Our finding of a latent interaction between schools and children’s EA-PGI lays the foundation for further work identifying *how* schools magnify or suppress the effects of genetic differences between children on their achievement. More detailed facets of children’s experiences of school may account for part of the latent interaction. Understanding how schools differentiate students with similarly low EA-PGI may help to identify social barriers to be removed through policy. This is an exciting prospect given the difficulties involved with identifying interventions via randomised controlled trials and other designs^33^. Future studies should also investigate within-school interactions with children’s genetics. Indeed, the similarity between Norwegian schools leaves room for important within school effects of teachers and friends. Additionally, family investments and educational support might moderate school effects.

In sum, social influences on academic achievement are theorised to be multilevel and interactive. In a large population-wide sample we see that schools but not residential environments (which only have small effects on achievement) interact with students’ EA-PGI. This social-genetic approach is necessary for a complete understanding of how children’s social environments work, and how to reduce school-driven differences in achievement between children with otherwise similar individual characteristics.

## Methods

### The Norwegian context

Norway is a wealthy social democratic welfare state^34^ with low unemployment and relatively low-income inequality compared to other wealthy nations^35^. Nonetheless, wealth inequality^36^ and child poverty are substantial, and exacerbating over time^37^. With respect to Norway’s education system, the public sector at the municipality level is responsible for providing various welfare services, including (free) compulsory education. Compulsory education is comprehensive with a common curriculum for all students, and there is no tracking. Fewer than 4% of students attend private schools, which are mainly schools with alternative pedagogical traditions, religious schools, or international schools. With respect to residential patterns, most elementary school children attend their local public school.

### Sample

The Norwegian Mother, Father and Child Cohort Study (MoBa^38^) is a prospective population-based pregnancy cohort study conducted by the Norwegian Institute of Public Health. Pregnant women were recruited from across Norway from 1999 to 2009. The women consented to initial participation in 41% of the pregnancies. Of fathers invited to participate, 82.9% consented. Parents consented on behalf of children. The total cohort includes approximately 114,500 children, 95,200 mothers and 75,200 fathers. To date, 98,110 individuals who are part of a trio (both parents and a child) from MoBa have been genotyped.

The present analyses were conducted on a subsample of parent-offspring trios with complete data for genome-wide genotyping, and administrative records of educational achievement, school, neighbourhood, district, and municipality membership, linked to MoBa through the Norwegian national ID number system. The administrative data are of high quality, and do not suffer from attrition^39,40^. Prior to analysis, we restricted the sample to one child per family, choosing one sibling at random. We also restricted the sample to those with complete register data on parental education and income. In further analyses, we also restricted the sample to those with complete register data on school sociodemographics.

### Ethics

The establishment of MoBa and initial data collection was based on a licence from the Norwegian Data Protection Agency and approval from The Regional Committees for Medical and Health Research Ethics. The MoBa cohort is now based on regulations related to the Norwegian Health Registry Act. The current study was approved by The Regional Committees for Medical and Health Research Ethics (project # 2017/2205).

### Measures

#### School achievement

Standardised national test results for maths and reading at grades 5, 8, and 9, and English at grades 5 and 8 were obtained through linkage to Norway’s National Education Database. Introduced in 2007, these tests are mainly used to monitor school development over time. Tests are compulsory, with 96% of all students in Norway taking them; students with special needs and those following introductory language courses may be exempt. Results are conveyed to teachers and parents but have no direct consequence for students. We residualised students’ test scores for sex, current age (to capture birth cohort effects), and the exact age when they took the tests. We created ‘core achievement’ measures as mean scores at each grade across available subjects and centred these to have mean zero, and standard deviation one. Our prior study showed that the standardised test outcomes are approximately normally distributed, with no indication of skewness or of ceiling effects, and were strongly correlated with item-response theory-derived scores^21^.

#### School, neighbourhood, district, and municipality identifiers

We matched children’s achievement results to the schools they attended and areas they lived in when they took each test. School identifiers were obtained from the National Education database (NUDB), and identifiers for three levels of residential information (neighbourhoods, districts, and municipalities) were obtained from the Norwegian central population register. Neighbourhood identifiers are for basic statistical units, called *grunnkretser*, which were designed by Statistics Norway to cover consistent numbers of inhabitants (~350) living in homogeneous conditions. Neighbourhoods are nested within larger *delområde*, or districts, which are in turn nested within municipalities (known as *kommune* in Norwegian). Children from one neighbourhood sometimes attend different schools, and children attending the same school may live in different neighbourhoods.

Importantly, to harmonise the identifiers such that students who resided nearby were identified as such, we converted as many identifiers as possible to match 2018 values. We chose 2018 since this is the most recent time-point covered by our linked administrative data. The majority of the changes between 2011-18 (the years that MoBa children took the standardised national tests) were merges of nearby municipalities and districts, and are summarised here: https://www.ssb.no/metadata/alle-endringer-i-de-regionale-inndelingene. For example, in 2018, 0702 Holmestrand and 0714 Hof slått municipalities were merged to create 0715 Holmestrand. We therefore changed any occurrences of 0702 and 0714 to 0715. After merging to 2018 boundaries, the total number of municipalities in our analysis sample reduced from 460 to 408. We also performed our analyses using the original residential identifiers (before harmonising to 2018 values), and reached the same conclusions (same best-fitting model, with almost identical estimates; see Supplementary Tables 5a–e). Note that it was not possible to use 2018 values for areas that were split into new identifiers for smaller areas. For example, in 2017, Oslo’s Grønland district 1 was split into three new areas (Grønland 7, 8 and 9), but it is unknown which of the three sub-identifiers should be given to students for 2011-16. Also note that changes involving the most fine-grained level (neighbourhood) were not possible to harmonise, because these, like school identifiers, were anonymous. These limitations mean that for a minority of residential areas we can only detect within-cohort area effects on achievement.

#### Sociodemographic measures for schools and residential areas

To complement the latent analyses, we tested whether specific sociodemographic measures could explain interactions identified through multilevel modelling. We created sociodemographic measures by aggregating administrative data from all parents of students at each school with register data, not only MoBa participants. Since interactions were solely present at the school-level, we did not test measures aggregated to residential areas. Measures were intended to capture both the average sociodemographic background among students within each school, and the variability of sociodemographic backgrounds of students within each school. For each school, we included five measures. The first measure was the average years of completed education of parents, converted from Norwegian Standard Classification of Education (NUS2000) categories, and measured when students were 16. The second sociodemographic indicator was the average parental pre-tax annual income from gainful employment including self-employment but not capital income or social welfare transfers. We averaged the income of both parents across the years that children were aged 11-15, and ranked their income compared to other parents in the same birth cohort. Third and fourth, we measured socioeconomic inequality by calculating Gini coefficients in reported levels of parental education and income, respectively. Gini is a widely used single measure of inequality, and ranges from 0 to 1, with 0 indicating absolute equality and 1 indicating absolute inequality. Fifth, we calculated the proportion of children who are non-Western immigrants and/or who are the children of non-Western immigrants. We created these broad measures in the absence of more detailed school data. Notably, the measures could capture effects intrinsic to specific schools (e.g., peer effects) or broader social stratification (e.g., composition of the school catchment area). If the latter is true, then these variables could be considered additional controls for selection into schools and neighbourhoods.

We used the same measures of parental educational attainment and earned income as individual-level control variables in all analyses.

### Genotype quality control

The current MoBa genomic dataset comprises imputed genetic data for 98,110 individuals (~32,000 parent-offspring trios; before quality control), derived from nine batches of participants, who make up four study cohorts. Within each batch, parent and offspring genetic data were quality controlled separately. Pre-imputation quality control criteria have been described in previous publications. We conducted post-imputation quality control, retaining SNPs meeting the following criteria: imputation quality score ≥ 0.8 in all batches, non-duplicated (by position or name), call rate >98%, minor allele frequency >1%, Hardy-Weinberg equilibrium p < 0.001, not associated with genotyping batch at the genome-wide level, and not causing a mendelian error. We removed individuals with the following criteria: heterozygosity outliers (F-het + /− 0.2), call rate <98%, reported sex mismatching SNP-based sex, duplicates (identified using PLINK’s^41^ –genome command as having pihat > =0.98, and distinguished from monozygotic twins through linkage to unique IDs in the population register, plus age, sex, and kinship information within MoBa), individuals with excessive numbers of close relatives (cryptic relatedness) and mendelian errors. To minimise environmental confounding, we identified a sub-sample of individuals with European ancestries via principal component analysis using the 1000 Genomes reference; thresholds for exclusion of outliers were based on visual inspection of a plot of principal components 1 and 2. The final numbers of individuals and SNPs passing quality control were 93,582 and 6,797,215, respectively. Principal components of genetic ancestry were computed for all participants using PLINK’s –within and –pca-clusters commands, based on an LD-pruned version of the final quality-controlled genotype data.

### Educational attainment polygenic index (EA-PGI)

We generated EA-PGI for all 93,582 parents and children in MoBa who passed quality control, based on genome-wide association summary statistics^42^ excluding 23andMe and MoBa samples. We used the PRSice software to calculate scores using all SNPs (i.e., p-value threshold of 1), with clumping parameters kb = 500, *p* = 1, r2 = 0.25. We computed mid-parental PGI by taking the average maternal and paternal PGI. PGI for children from independent families and mid-parental PGI (hereafter ‘parental PGI’) were then centred to have mean zero, and standard deviation one. In all PGI analyses, we included parental PGI as controls, such that effects of offspring PGI are within-family direct genetic effects. We also included principal components (5 based on maternal data, 5 based on paternal data) to control for population stratification in the parental EA-PGI effects.

The advantage of the within-family EA-PGI is that it controls for non-random selection of schools by parents. We conducted a proof-of-concept test of this by quantifying the degree of clustering of children’s EA-PGI in schools. The intraclass correlation coefficient indicated that schools capture 2.6% of the variance in the child EA-PGI. The child EA-PGI was clustered even less in residential areas (ICCs for at neighbourhood, district and municipality were 0.3%, 0.1%, and 1.2%, respectively; Supplementary Table 4b). However, once parent EA-PGI is adjusted for, 0% of the variance in within-family child EA-PGI is explained by schools. This implies that conditional on parental EA-PGI, the sorting of students into schools is random and we can interpret the school slopes based on the within-family PGI causally. Note that the degree of clustering of genetic risk in schools is likely to be larger than estimated using the EA-PGI, which only explains ~2-8% of the variance in child achievement.

Notably, although we treat parental EA-PGI as control variables, they allow us to estimate a parental indirect genetic effect. This represents an environmental effect of parents’ education-linked genetics on the child’s achievement. However, parental indirect genetic effects, and their moderation, capture selection into schools and residential areas and may be biased by population stratification, assortative mating, and passive gene-environment correlation (unlike the within-family child genetic effect).

### Statistical analyses

To test for interactions of individual genetic propensity for educational attainment with schools and residential areas, we compared a series of increasingly complex multilevel models (11 in total). To ensure that findings were not simply produced by chance, we formally compared AIC fit statistics.

The base model (Model 1) estimated the association between achievement and the within-family EA-PGI (child PGI controlling for mid-parent PGI). We pooled data across grades by including individual identification number as a random intercept, and time-point as a fixed effect to account for mean differences in scores across time. Time-point was coded as a continuous variable centred with 0 for grade 9, -1 for grade 8, and -4 for grade 5. Note that the grade 9 composite only includes maths and reading, whereas achievement composites for grades 5 and 8 include maths, reading and English.

In Models 2a-d, we tested the degree to which achievement varied between social contexts. We started with achievement variation at only the most proximal level (school; Model 2a), and eventually allowed for context effects at all levels (school, neighbourhood, district, and wider municipality; Model 2d). Specifically, we added random intercepts for schools and residential areas in multilevel regression models. Residential clusters are nested, with neighbourhoods sitting within districts, and districts sitting within municipalities. Since children living in one area can attend different schools, and schools contain children living in multiple areas, schools are cross classified with the residential clusters.

In Models 3a-d, having established the best-fitting pattern of contextual stratification of achievement, we used random slope models to estimate the extent that contexts interact with EA-PGI effects. In our models, we allowed PGI effects to vary for each cluster with significant intercept variance and tested whether model fit improved.

### Environments explaining the variability of slopes

To investigate which characteristics explaining any gene-environment interactions, we re-estimate the best-fitting multilevel model for each school subject, adding fixed effects for five environmental measures (Model 4), and then environment-by-PGI interaction terms (Model 5). School environmental influences on achievement are likely multifactorial, operating in a ‘poly-environmental’ mode akin to polygenicity. We therefore included measured environments simultaneously. If measured environments account for an interaction, the variance in slopes will be reduced and model fit will be improved in Model 5 compared to Model 4. The five sociodemographic measures were tested jointly. Notably, while we term these observed measures ‘environments’, they are themselves partially under genetic influence.

### Model-fitting and comparisons

In all models, 10 principal components of genetic ancestry were included as covariates to control for population stratification, 5 based on maternal genotype and 5 paternal components. Although effects of the child EA-PGI are robust to population stratification (when parental scores are included), the inclusion of PCs helps us interpret the parental genetic effect, which may be biased by population stratification. All models included controls for family social background (parental education and income), to aid causal interpretation of slopes and intercepts for schools and residential areas.

Models were compared using the AIC fit statistic, which calculates the trade-off between model fit and model complexity using maximum likelihood modelling with a penalty for the number of parameters. If the model with, for instance, the random slopes across schools, has a lower AIC value than that of a simpler model, this is evidence that gene-environment interactions should be included for an optimal approximation of the underlying data generating processes. We also report p-values from the model comparison tests.

### Software

Maps were created with the R package fhimaps^43^, using the 2019 municipality boundaries (only 1 had to be changed from the 2018 identifiers used in our main analyses). Model-fitting was conducted in R with the lme4 package^44^.

## Supplementary information

Supplementary Material

## Data Availability

MoBa data are available to individuals who obtain the necessary permissions from the data access committee (see https://www.fhi.no/en/studies/moba/for-forskere-artikler/research-and-data-access/).

## Code availability

Analysis code can be found at https://github.com/rosacheesman/genes-schools-areas. All additional software used to perform the analyses are available online.

## References

[1] Bronfenbrenner, U. & Morris, P. A. In *Handbook of child psychology* (eds. Damon, W. & Lerner, R. M.) (John Wiley & Sons, Inc., 2007). 10.1002/9780470147658.chpsy0114.

[2] Galster, G. C. in *Neighbourhood Effects Research: New Perspectives* (eds. van Ham, M., Manley, D., Bailey, N., Simpson, L. & Maclennan, D.) 23–56 (Springer Netherlands, 2012). 10.1007/978-94-007-2309-2_2.

[3] Duncan GJ, Murnane RJ (2014). Growing income inequality threatens american education. Phi Delta Kappan.

[4] Nicoletti C, Rabe B (2013). Inequality in pupils’ test scores: how much do family, sibling type and neighbourhood matter?. Economica.

[5] Laliberté J-W (2021). Long-Term Contextual Effects in Education: Schools and Neighborhoods. Am. Economic J.: Economic Policy.

[6] Plomin R, DeFries JC, Loehlin JC (1977). Genotype-environment interaction and correlation in the analysis of human behavior. Psychol. Bull..

[7] Scarr-Salapatek S (1971). Race, social class, and IQ. Science.

[8] Harden, K. P. *The Genetic Lottery: Why DNA Matters for Social Equality*. (2021).

[9] Figlio DN, Freese J, Karbownik K, Roth J (2017). Socioeconomic status and genetic influences on cognitive development. Proc. Natl Acad. Sci. USA.

[10] Tucker-Drob EM, Bates TC (2016). Large Cross-National Differences in Gene × Socioeconomic Status Interaction on Intelligence. Psychol. Sci..

[11] Baier, T. et al. Genetic Influences on Educational Achievement in Cross-National Perspective. *Eur. Sociol Rev.*10.1093/esr/jcac014 (2022).

[12] Allegrini, A. G. et al. Multivariable G-E interplay in the prediction of educational achievement. *BioRxiv*10.1101/865360 (2019). 10.1371/journal.pgen.1009153 PMC7721131 33201880

[13] Selzam S (2017). Predicting educational achievement from DNA. Mol. Psychiatry.

[14] Isungset MA (2022). Social and genetic associations with educational performance in a Scandinavian welfare state. Proc. Natl Acad. Sci. USA.

[15] Harden KP, Turkheimer E, Loehlin JC (2007). Genotype by environment interaction in adolescents’ cognitive aptitude. Behav. Genet.

[16] Boardman JD, Daw J, Freese J (2013). Defining the environment in gene-environment research: lessons from social epidemiology. Am. J. Public Health.

[17] Taylor J, Roehrig AD, Soden Hensler B, Connor CM, Schatschneider C (2010). Teacher quality moderates the genetic effects on early reading. Science.

[18] Haughbrook, R., Hart, S. A., Schatschneider, C. & Taylor, J. Genetic and environmental influences on early literacy skills across school grade contexts. *Dev. Sci*. **20**, (2017). 10.1111/desc.12434 PMC5293682 27496364

[19] Hart SA, Soden B, Johnson W, Schatschneider C, Taylor J (2013). Expanding the environment: gene × school-level SES interaction on reading comprehension. J. Child Psychol. Psychiatry.

[20] Trejo S (2018). Schools as Moderators of Genetic Associations with Life Course Attainments: Evidence from the WLS and Add Health. Sociol. Sci..

[21] Cheesman, R. et al. How interactions between ADHD and schools affect educational achievement: a family‐based genetically sensitive study. *J. Child Psychol. Psychiat.*10.1111/jcpp.13656 (2022). 10.1111/jcpp.13656 PMC9796390 35789088

[22] D’Onofrio BM, Lahey BB, Turkheimer E, Lichtenstein P (2013). Critical need for family-based, quasi-experimental designs in integrating genetic and social science research. Am. J. Public Health.

[23] Belsky DW (2019). Genetics and the geography of health, behaviour and attainment. Nat. Hum. Behav..

[24] Keller MC (2014). Gene × environment interaction studies have not properly controlled for potential confounders: the problem and the (simple) solution. Biol. Psychiatry.

[25] van der Sluis S, Posthuma D, Dolan CV (2012). A note on false positives and power in G × E modelling of twin data. Behav. Genet.

[26] Hægeland T, Raaum O, Salvanes KG (2012). Pennies from heaven? Using exogenous tax variation to identify effects of school resources on pupil achievement. Econ. Educ. Rev..

[27] Hermansen AS, Borgen NT, Mastekaasa A (2020). Long-Term Trends in Adult Socio-Economic Resemblance between Former Schoolmates and Neighbouring Children. Eur. Socio. Rev..

[28] Harden KP (2020). Genetic associations with mathematics tracking and persistence in secondary school. NPJ Sci. Learn..

[29] von Stumm, S. et al. School quality ratings are weak predictors of students’ achievement and well-being. *J. Child Psychol. Psychiatry*10.1111/jcpp.13276 (2020). 10.1111/jcpp.13276 PMC8298902 32488912

[30] Falch T, Sandsør AMJ, Strøm B (2017). Do Smaller Classes Always Improve Students’ Long-run Outcomes?. Oxf. Bull. Econ. Stat..

[31] Wang H (2019). Genotype-by-environment interactions inferred from genetic effects on phenotypic variability in the UK Biobank. Sci. Adv..

[32] Smith-Woolley E (2018). Differences in exam performance between pupils attending selective and non-selective schools mirror the genetic differences between them. NPJ Sci. Learn..

[33] Bromann, K. Randomized Controlled Trials Commissioned by the Institute of Education Sciences Since 2002: How Man. *Policy Commons* (2013).

[34] Esping-Andersen, G. *The Three Worlds of Welfare Capitalism*. (1990).

[35] Eurofound. Annual review of working life. https://www.eurofound.europa.eu/publications/report/2018/annual-review-of-working-life-2017. (2017).

[36] Pfeffer, F. T. & Waitkus, N. The wealth inequality of nations. *Am. Sociol. Rev*. 000312242110278 (2021). 10.1177/00031224211027800.

[37] Barth, E., Moene, K. & Pedersen, A. W. In *Europe’s income, wealth, consumption, and inequality* 218–245 (Oxford University Press, 2021). 10.1093/oso/9780197545706.003.0006.

[38] Magnus P (2016). Cohort profile update: the norwegian mother and child cohort study (moba). Int. J. Epidemiol..

[39] Røed K, Raaum O (2003). Administrative registers – Unexplored reservoirs of Scientific Knowledge?. Economic J..

[40] Hovde Lyngstad T, Skardhamar T (2011). Nordic register data and their untapped potential for criminological knowledge. Crime. Justice.

[41] Chang CC (2015). Second-generation PLINK: rising to the challenge of larger and richer datasets. Gigascience.

[42] Lee JJ (2018). Gene discovery and polygenic prediction from a genome-wide association study of educational attainment in 1.1 million individuals. Nat. Genet..

[43] The easier way to create a map of Norway using {fhimaps} - Daniel Roelfs. https://danielroelfs.com/blog/the-easier-way-to-create-a-map-of-norway-using-fhimaps/.

[44] Bates D, Mächler M, Bolker B, Walker S (2015). Fitting linear mixed-effects models using lme4. J. Stat. Softw..

